# From Film Processing to Microphase Orientation: Structure–Property Relationships in Commercial PBSA/PLA Blend Films

**DOI:** 10.3390/polym18060761

**Published:** 2026-03-20

**Authors:** Guru Geertz, Stefan Böhler, Bastian Barton, Frank Malz, Andreas Bohn, Olaf Kahle, Robert Brüll, Jens Balko

**Affiliations:** 1 Division Plastics, Fraunhofer Institute for Structural Durability and System Reliability LBF, Schlossgartenstr. 6, 64289 Darmstadt, Germanyfrank.malz@lbf.fraunhofer.de (F.M.); robert.bruell@lbf.fraunhofer.de (R.B.); 2 Division Polymer Processing, Fraunhofer Institute for Applied Polymer Research IAP, Schipkauer Str. 1, A754, 01987 Schwarzheide, Germany; 3 Division PYCO, Fraunhofer Institute for Applied Polymer Research IAP, Schmiedestr. 5, 15745 Wildau, Germany

**Keywords:** bioplastic, blend, processing, flexible film, confocal Raman microscopy

## Abstract

The commercialization of poly(butylene succinate-co-adipate) (PBSA), a biodegradable and potentially fully biobased random copolyester, is still ongoing. Due to its high relevance as mono material or as blend component in flexible film applications, a sound understanding of compounding, further processing and film properties is necessary. In this work, PBSA, poly (lactic acid) (PLA) and blends at three different compositions thereof were processed into flat films and blown films, respectively. Investigating the films with X-ray diffraction (XRD), multivariate confocal Raman microscopy (CRM) and scanning electron microscopy (SEM) revealed the semicrystalline order as well as the blend morphology. While PBSA is semicrystalline, PLA remains amorphous after the processing step. As imaged by CRM, flat films exhibit lamellar-like domains formed during uniaxial stretching and rapid cooling, whereas blown films show no pronounced preferential orientation. Tensile tests in both the machine and transverse directions demonstrate the versatility of PBSA and its blends in spanning a wide range of mechanical strength and flexibility, covering and partly exceeding the stiffness and strength ranges typically reported for commodity polyolefins while exhibiting reduced ductility. Differential scanning calorimetry (DSC) and dynamic mechanical analysis (DMA) provide further insights into the thermal properties of the pure and blend materials.

## 1. Introduction

Poly(butylene succinate) (PBS) and its random copolymers poly(butylene succinate-co-adipate) (PBSA) are biodegradable aliphatic polyesters that have attracted considerable interest as potential alternatives to petro-based polyolefins in flexible thermoplastic applications such as flat films, blown films, thermoforming, blow molding, and injection molding.

PBS and PBSA can be synthesized by polycondensation using monomers from petrochemical or renewable resources, and their biodegradability in soil and aqueous environments has been well established [1,2]. Industrial production routes for biobased succinic acid, 1,4-butanediol, and adipic acid are available, enabling partially or fully biobased PBSA grades [3,4,5].

PBSA is a semicrystalline copolyester in which succinate and adipate units are randomly distributed along the polymer chain [6]. Its melting temperature, glass transition temperature, and degree of crystallinity depend strongly on copolymer composition. Whereas PBS melts at approximately 115 °C, PBSA with higher adipate content exhibits significantly reduced melting and glass transition temperatures [1,7,8]. Wide-angle X-ray diffraction studies revealed an isodimorphic crystallization behavior, with a transition from PBS-like to PBA-like unit cells at intermediate adipate contents and partial comonomer inclusion into the crystalline lattice [8].

Commercial PBSA grades intended for industrial processing typically contain 20–30 mol % adipate units, providing a balance between processability, thermal stability, mechanical performance, and biodegradability. Early commercial materials such as Bionolle were extensively studied with respect to molecular architecture and crystallization behavior [9,10]. More recently, partially biobased PBSA grades marketed under the name BioPBS have become available. Their processing behavior and thermal stability under industrial extrusion conditions were reported to differ from earlier PBSA materials, highlighting the relevance of studying commercially available grades rather than model polymers [11]. Although PBS and PBSA already exhibit mechanical and thermal properties comparable to those of commodity polyolefins, their performance can be tailored by blending with other biodegradable polyesters. In particular, blends of PBS or PBSA with poly(lactic acid) (PLA), the most widely used biobased polyester, have been investigated in numerous studies [12,13,14]. PBSA/PLA blends are generally immiscible, and their phase morphology strongly depends on blend composition and processing history [15,16]. For compression-molded specimens, dispersed spherical domains of the minor phase with typical sizes in the micrometer range have been reported [15,17].

Beyond conventional microscopy techniques, confocal Raman microscopy (CRM) has emerged as a powerful tool to investigate the spatial distribution and orientation of polymer phases in immiscible blends. Raman studies on PLA/PBS systems demonstrated that quiescent blends are largely isotropic, whereas pressure- or flow-induced processing can lead to pronounced molecular orientation [18]. CRM has also been applied to other immiscible polyester blends, such as PLA/PCL, to resolve composition-dependent phase morphologies [19]. However, many CRM studies rely on univariate spectral analysis, which may be insufficient for complex or finely structured multiphase systems. Recent work has shown that multivariate analysis techniques significantly enhance the interpretability of Raman data, enabling quantitative phase mapping in heterogeneous polymeric and soft-matter systems [20,21].

Despite the extensive literature on PBSA, PBSA/PLA blends, and Raman-based characterization methods, systematic studies addressing commercial PBSA/PLA blends processed into thin flat and blown films under industrially relevant conditions remain scarce. In particular, the combined effects of film processing, microphase orientation, semicrystalline structure, and direction-dependent mechanical behavior have not been comprehensively investigated. In this work, commercially available PBSA and PLA are blended and processed into flat and blown films. The resulting morphology, crystalline structure, thermal behavior, and mechanical properties are analyzed with a special focus on processing-induced orientation, employing confocal Raman microscopy combined with multivariate data analysis.

## 2. Materials and Methods

### 2.1. Materials and Compounding

Commercially available polymers were supplied as food contact-grade poly (butylene succinate-co-adipate) (BioPBS FD92PM) by PTT MCC Biochem, Map Ta Phut, Thailand and poly (lactic acid) (Ingeo 4043D) by NatureWorks LLC, Plymouth, MI, USA, respectively, both in the form of granules. The PBSA grade is suitable for film extrusion, having a melt flow index of 4 g/10 min (2.16 kg, 190 °C) and a melting point of 88 °C. The number-averaged molar mass $M_{n}$ = 32 kg/mol and dispersity D = 5.7 as determined by size exclusion chromatography (SEC) are reported elsewhere [22]. The PLA grade Ingeo 4043D is a multipurpose extrusion grade with a melt flow index of 2.5 g/10 min (2.16 kg, 190 °C), a level of D-isomer of 4 to 5 wt.% and a peak melting temperature between 145 and 160 °C according to information by the manufacturer. The SEC analysis yielded $M_{n}$ = 74 kg/mol and D = 2.9 [22].

Prior to compounding, the granules were stored in a dry-air dryer GTT 101 ES (Gerco, Warendorf, Germany) at 40 °C for at least 4 h. Blending of the polymeric components was performed on a co-rotating twin-screw extruder ZSE 27MAXX (Leistritz Extrusionstechnik GmbH, Nurnberg, Germany) with a screw diameter D = 28.3 mm and a L/D = 52, where L is the length of the screw. The blend compositions were PBSA/PLA = 80 wt.%/20 wt. % (abbreviated PBSA-80), 50/50 (PBSA-50) and 20/80 (PBSA-20). The extruded strand was cooled in water, dried under compressed air on a conveyor belt and pelletized. According to the blend composition, the extrusion parameters varied in speed of rotation *n* = 300…350 rpm and melt temperature $T_{melt}$ = 193 °C to 213 °C at the extruder die, respectively.

The compounded PBSA/PLA blends were pelletized and subsequently used as feed material for all flat film and blown film extrusion experiments.

### 2.2. Film Processing

Flat films (FF) and blown films (BF) were produced from virgin PBSA, PLA, and the compounded PBSA/PLA blends. Although a three-layer flat film line was used, all films were produced as monolayer films by feeding identical material to all extruders. A three-layer flat film line from Collin Lab & Pilot Solutions GmbH, Maitenbeth, Germany, was used and equipped with three single-screw extruders (Collin, D = 25 mm, L/D = 25), a flat die with 250 mm width and a chill-roll unit with pull-off. The flat die height was set to 0.3 mm. The extruders operated at n = 30–50 rpm. After extrusion from the flat die, the film was cooled on the chill-roll to a temperature of 40 °C. The final flat film width after pull-off was between 130 and 160 mm. Blown films were produced with a single-screw extruder (Collin, D = 25 mm, L/D = 25), a ring die of diameter $D_{die}=\;$50 mm and 0.8 mm slit width followed by layflat and withdrawal. The widths of the blown films ranged from 120 to 140 mm. Melt temperatures in the flat and ring die during extrusion were adjusted according to stable processing conditions and film quality. The resulting film thickness $t_{film}$ and surface roughness $R_{a}$ were determined in the transverse direction using a micrometer gauge and profilometry (Dektak 8, Veeco, Plainview, NY, USA). The surface roughness was determined from three single measurements within a lateral scan range of 0.3 mm. A compilation of melt temperatures at the die $T_{melt}$, $t_{film}$ and $R_{a}$ is given in Table 1.

The significantly increased surface roughness observed for the PBSA/PLA blend films compared to the neat polymers (Table 1) can be attributed to the immiscible blend morphology in combination with rapid solidification during film extrusion. While neat PLA remains amorphous and neat PBSA forms a comparatively homogeneous semicrystalline surface, the blends exhibit phase-separated microstructures with different crystallization and relaxation behavior of the individual components. In particular, crystallization of the PBSA phase during cooling, combined with the suppressed relaxation of the immiscible blend morphology under high cooling rates typical for thin film extrusion, leads to a pronounced surface relief. This effect is more pronounced for flat films, where uniaxial stretching and rapid quenching further promote frozen-in surface topography

### 2.3. Characterization

#### 2.3.1. Multivariate Confocal Microscopy

Confocal Raman maps of blend films were recorded using an Alpha 500 Raman microscope (WITec, Ulm, Germany) combined with a 532 nm Ne:YAG laser with power adjusted to 10 mW. Rectangular samples of ~2 × 1 cm were cut out from the flat film and fixated on the bottom of a small glass Petri dish, which was then filled with de-ionized water. A 63× 1.0 NA water immersion objective (Zeiss, Oberkochen, Germany, W Plan-Apochromat 63×/1.0 M27) was then used for Raman scanning to increase optical resolution, and to reduce thermal degradation of the foil during acquisition by heat dissipation in the surrounding water. Optical focus was placed ~10 µm below the foil surface to compensate for the surface roughness. An area of 100 × 100 µm was scanned in the XY plane using a sampling of 100 × 100 spectra, with 500 ms integration time for each spectrum. The resulting 10,000 Raman spectra per map were corrected for fluorescent background by asymmetric least squares smoothing [23], reduced to a spectral region of interest from 500 to 3300 cm^−1^, after which vector normalization was applied.

Raman datasets obtained from flat films and blown films at five different PBSA/PLA compositions each were jointly used to fit a non-negative matrix factorization (NMF) model implemented in Python 3.9.16 using the Scikit-Learn 1.2.1 library [24] with two components. The trained NMF model was subsequently applied to all individual datasets to decompose the Raman maps into identical component spectra and corresponding concentration maps. Finally, the resulting concentration maps for each component were noise-reduced using nonlinear anisotropic (Perona-Malik) diffusion with n_iter_ = 1, κ = 50 and γ = 0.1. All data processing routines were implemented in Python 3.9.16.

#### 2.3.2. Scanning Electron Microscopy (SEM)

A Topcon SM300 scanning electron microscope (Topcon, Tokyo, Japan) operating at 6 kV was used to investigate the morphology of the fracture surface of the PBSA/PLA films. The films were fractured under cryogenic conditions using liquid nitrogen. Subsequently, the specimens were sputtered with gold.

#### 2.3.3. X-Ray Diffraction

A Bruker-AXS Kristalloflex 760 X-ray generator and a flat-film camera (Freiberger Präzisionsmechanik, Freiberg, Germany) were used to carry out wide-angle X-ray diffraction (WAXS) experiments. The generator was operated at 40 mA and 40 kV. The X-rays with a wavelength of 0.15418 nm were monochromized by a Ni-filter. The PBSA/PLA film samples were oriented with the face perpendicular to the primary X-ray beam and were aligned vertically with the long side of the films (machine direction). The diffraction patterns were recorded by a flat film (Biomax, Kodak, Rochester, NY, USA), which was exposed for 2 h. The sample-to-film distance was 50 mm. To estimate the crystalline orientation semi-quantitatively, the azimuthal intensity profiles along the Debye-Scherrer rings on the scanned X-ray films were plotted using the public image analysis software ImageJ 1.49u. The (020) lattice planes at 2θ: 19.5° of PBSA were used for evaluation. From the distribution peaks, the half-widths (FWHM) were determined and an axial orientation degree OD_(hkl)_ was calculated according to: OD_(hkl)_: (180° − FWHM)/180°. The values for OD_(hkl)_ run from 1 (perfect orientation) to 0 (no orientation).

#### 2.3.4. Differential Scanning Calorimetry (DSC)

DSC measurements were performed with a DSC 204 F1 Phoenix^®^ (NETZSCH, Selb, Germany) equipped with an intracooler accessory. The experiments were performed under an N_2_ atmosphere, and the equipment was calibrated using tin and indium. First heating scans from −50 °C to 220 °C at a rate of 10 K/min were followed by cooling to −50 °C and a second heating to 220 °C.

#### 2.3.5. Dynamic Mechanical Analysis

DMA measurements were performed using an RSA-3 dynamic mechanical analyzer (TA Instruments, Seoul, Republic of Korea) with an intracooler. Measurements were performed in tension geometry (sample width 12 mm, free length 20 mm, thickness about 0.1 mm) with a heating rate of 4 K/min and a strain of 0.01% from −65 °C up to the temperature of melting of the film.

#### 2.3.6. Tensile Measurements

Tensile tests were conducted according to ISO 527-3 [25] using film specimens.

The tensile tests were performed with a universal material testing machine Z010 (Zwick/Roell, Ulm, Germany) by using a load cell of 1 kN. The testing speed for the determination of the E-modulus $E_{t}$ was 1 mm/min for 0.05% to 0.25% strain. For higher strains, the testing speed was 100 mm/min. Prior to testing, the films were cut to specimens having a width of 10 mm and stored at 23 °C/50% relative humidity for more than 16 h. Each material was tested using 10 specimens.

## 3. Results and Discussion

### 3.1. Blend Morphology and Semicrystalline Structure

**Figure 1:**
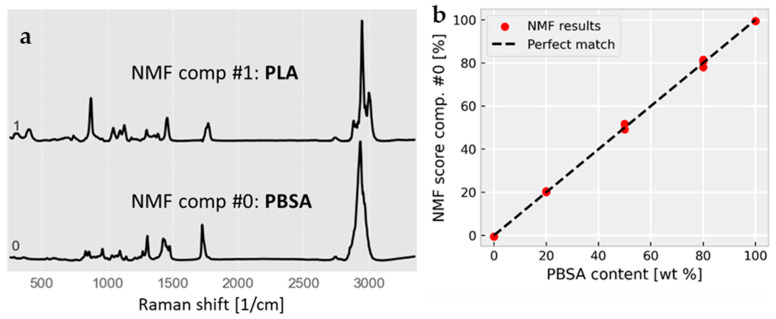
(**a**) Multivariate decomposition of confocal Raman spectra by non-negative matrix factorization (NMF) yields the Raman spectra of the components PBSA (#0) and PLA (#1). The decomposed data set contained Raman spectra from flat films and blown films of PBSA/PLA blends with five different compositions (0, 20, 50, 80, 100 wt % PBSA). (**b**) Mean NMF scores of PBSA obtained from multivariate decomposition of Raman maps of samples A–E (cf. Figure 2). An error plot for the PBSA content prediction by multivariate Raman mapping with a mean error of 3.8 wt %.

**Figure 2:**
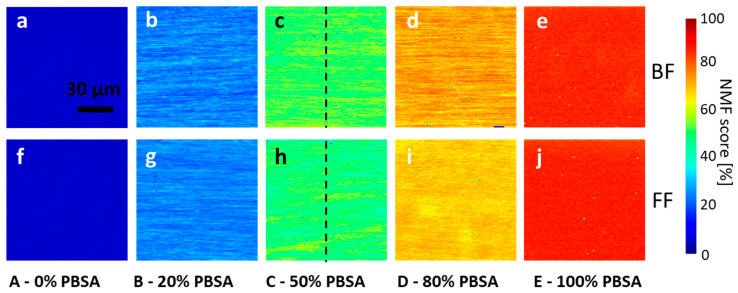
Decomposition of Raman spectra from films with five different compositions (0, 20, 50, 80, and 100 wt% PBSA) resulted in Raman maps of PBSA. The top row shows blown films (**a**–**e**), and the bottom row shows flat films (**f**–**j**). All images were generated using the same lookup table, shown as the color bar on the right. The normalized relative concentrations shown represent the NMF score and are not identical to chemical *w*/*w* concentrations. The black dashed lines in panels c and h indicate the positions along which the line profiles shown in Figure 4 were extracted. The physical image size of all maps is 100 × 100 μm, with a pixel size of 1 µm.

*Multivariate confocal Raman microscopy:* Conventional thermoplastic processing on a pilot-plant scale served as an industrially relevant method to prepare flat films and blown films to study their resulting film properties. The morphology of these PBSA/PLA films was investigated by confocal Raman microscopy on a micro scale, combined with multivariate hyperspectral processing. The results are shown in Figure 1 and Figure 2.

**Figure 3:**
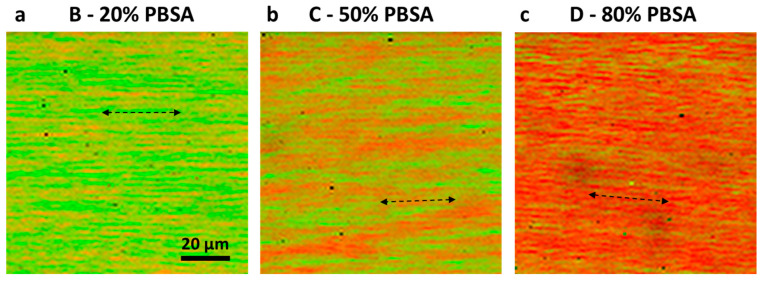
Overlays of NMF scores for flat films from PBSA (red) and PLA (green) obtained by multivariate Raman mapping (cf. Figure 2g–i). All three samples show a lamellar blend morphology whose orientation coincides with the machine direction of extrusion, indicated by dashed double-headed arrows.

Overlays of the NMF concentration maps for PBSA (Figure 2) indicate a directed, lamellar morphology for all the PBSA/PLA flat films and blown films (Figure 3).

The lamellar microphase morphology revealed by confocal Raman microscopy is consistent with the increased surface roughness of the PBSA/PLA blend films, suggesting that the phase-separated bulk morphology is at least partially reflected at the film surface.

**Figure 4:**
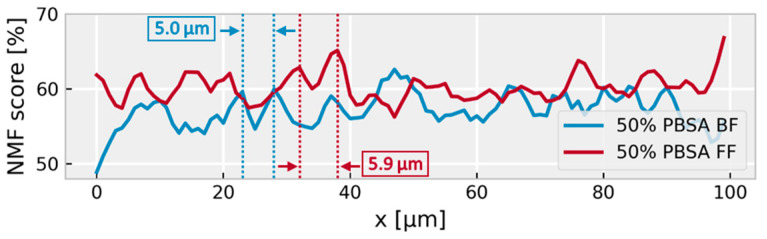
Line profiles of the PBSA Raman maps on the surfaces of samples PBSA-50-BF and PBSA-50-FF (cf. Figure 2), extracted along the black dashed lines in Figure 2c,h, respectively. The dashed vertical lines indicate adjacent maxima used as an example for estimating a local interlamellar distance; the illustrated values are 5.0 µm for BF and 5.9 µm for FF. In contrast, the reported mean interlamellar distances were calculated from the full profiles and are 6.6 ± 1.8 µm (BF) and 7.9 ± 3.1 µm (FF).

The decomposition of the Raman maps covering a square area of 100 × 100 µm with 1 µm spatial sampling resulted in two components, corresponding to PBSA and PLA as determined by a database search (Figure 1a). The PBSA component could not be further decomposed by the applied factor method, which is expected since the BS:adipate ratios of both samples are almost identical (~3:1, cf., samples PBSA-20-FF and PBSA-50-FF), and therefore these sub-components are not independent. All the PBSA concentration images of the PBSA/PLA blends show an oriented, lamellar blend morphology with typical interlamellar distances of ~7 µm for both the blown films and the flat films made of PBSA-50 (Figure 4). Note that the lamellar morphology observed on this length scale represents a property of the film bulk material, since the optical focus was set 10 µm below the film surface and the detection volume is about 1 µm^3^.

In contrast to the bulk or compression-molded PBSA/PLA blends reported in the literature, the observed lamellar morphology reflects processing-induced orientation under rapid cooling conditions typical for thin film extrusion.

**Figure 5:**
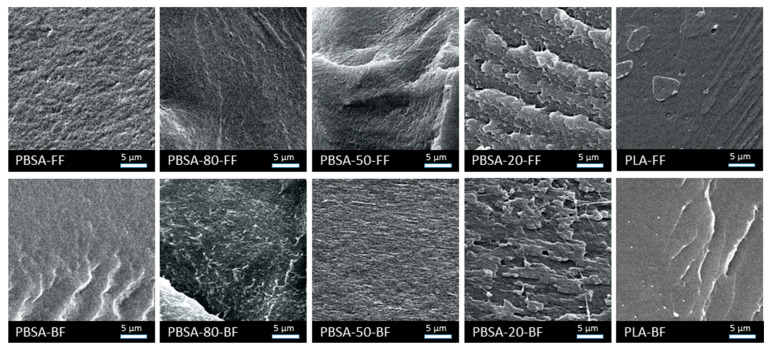
SEM images of the fracture surface of PBSA/PLA flat films (**upper row**) and blown films (**lower row**). The magnification is 2000×.

*Scanning Electron Microscopy*: To complement the insights obtained from confocal Raman microscopy, SEM measurements were performed on cryo-fractured surfaces of the flat films (FF) and the blown films (BF) (Figure 5). These SEM images show a variety of different fine structures, including both smooth fracture surfaces and more irregular topographies. Only the sample PBSA-20 FF exhibits a distinct lamellar-like morphology that matches the findings of CRM. The variations observed might be due to the cryogenic sample preparation, leading to a heterogeneous spatial pattern of the resulting fracture surface. Typically, fracture surfaces exhibit at least three distinct zones (origin, progression, overload) [26]. Hence, the different SEM images of a sample’s fracture surface depend on the region of interest.

The SEM images reveal, however, rougher surfaces of the pure PBSA films compared to the pure PLA. This is due to the crystallinity of the PBSA in contrast to the amorphous PLA. It is interesting to note that the fracture surfaces of PBSA/PLA films do not exhibit spherical morphologies as published by [27] A possible explanation for the deviating fracture surface morphology lies in the different processing of the blend specimens. The PBSA/PLA blends were injection molded into test bars (thickness 4 mm) in [27]. Under these conditions, the cooling time should be sufficient for a rearrangement of the sheared polymer into a spherical morphology. In this study, however, thin films (approx. 75 µm) were extruded, where rapid cooling leads to a frozen-in lamellar-like morphology due to shear. The SEM results obtained here—with minor evidence of lamellar morphologies in the PBSA/PLA films—are thus in agreement with those from Raman microscopy. As a drawback of the SEM approach, the blend morphology of the PBSA/PLA films is superimposed with the inherent features of the cryo-fractured surfaces, leading to a broad variety of experimental fine structures.

**Figure 6:**
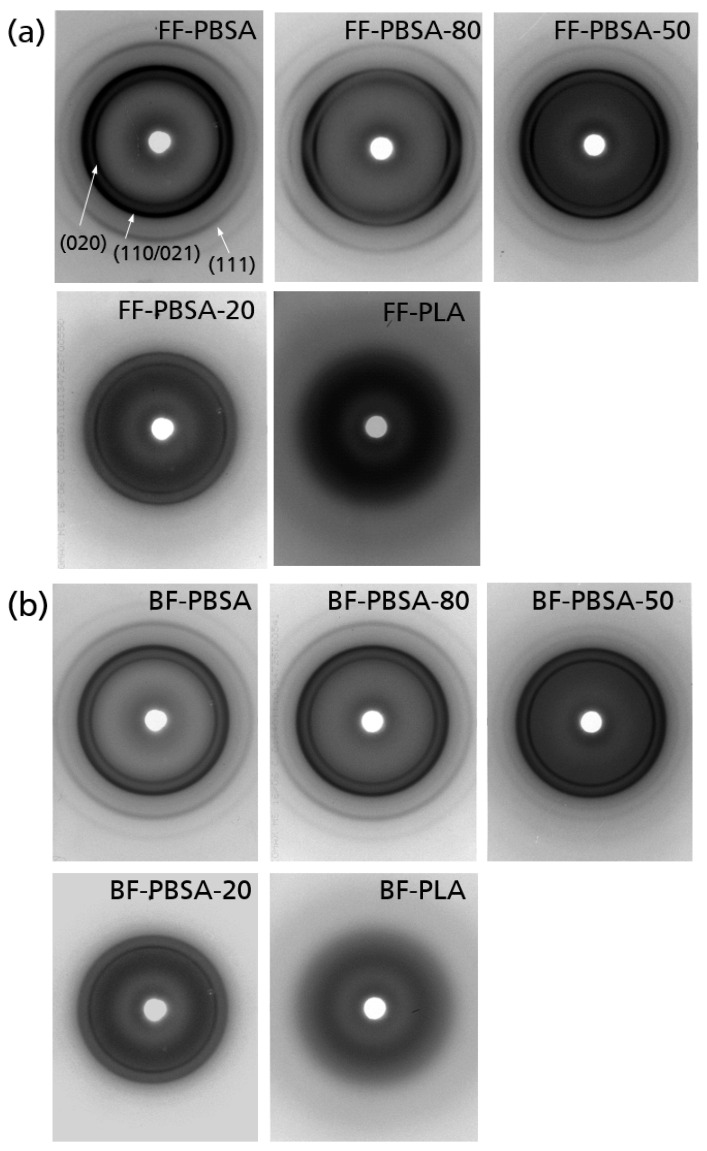
X-ray diffraction patterns in transmission geometry of the (**a**) flat films and (**b**) blown films.

*X-ray diffraction*: The XRD patterns detected in transmission geometry show the resulting semicrystalline order (Figure 6). Several sharp Bragg reflections resulting from the crystalline regions in PBSA show up in pure PBSA flat and blown films. The main reflections can be assigned to the Miller indices (020), (021), (110), (111) at 2θ-angles, which were 19.5°, 21.7°, 22.4° and 28.3°, respectively. In contrast, for poly (lactic acid) (PLA), only a broad and diffuse scattering pattern was detected, which is called an amorphous halo. The diffraction patterns of the blends are a superposition of the patterns of the pure materials. Whereas PBSA is crystallized in all the blends for flat and blown films, PLA remains in the amorphous, non-crystalline state. For PBSA-80-FF, the concentrated scattered intensity in equatorial arcs indicates an orientation of the crystals along the machine direction of the flat films (axial orientation degree OD_(020)_: 0.74), which corresponds to a medium orientation degree. An axial orientation is weaker but still visible for PBSA-50-FF (OD_(020)_: 0.71). The samples PBSA-20-FF and PBSA-100-FF show a very weak orientation, which, however, is not suitable for evaluation. For blown films, there are no such signatures of preferred crystalline orientation. This is reasonable, since here, the melt undergoes biaxial stretching in the machine and transverse direction. Blow up ratios of ~1.8 were realized for the PBSA containing the blown films produced in this work (defined by the ratio of the diameter of the bubble to the diameter of the ring die).

This clearly demonstrates that uniaxial stretching during flat film extrusion induces preferential crystalline orientation, whereas biaxial deformation during blown film processing suppresses directional crystallite alignment.

### 3.2. Mechanical Characterization

**Figure 7:**
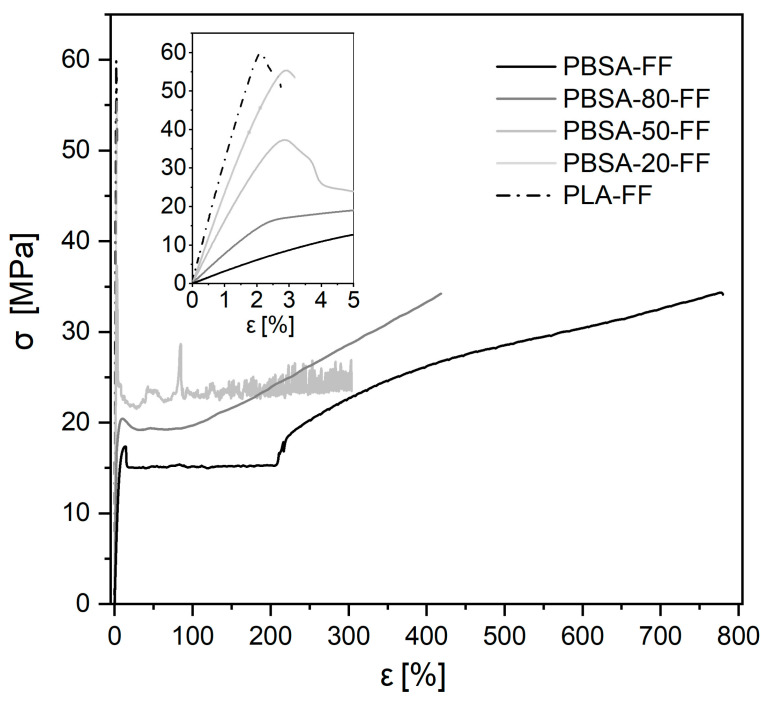
Exemplary stress–engineering strain curves from tensile measurements for the flat films in machine direction.

Tensile tests served to probe the quasi-static mechanical performance of the films. In Figure 7, the σ–ε curves (stress–engineering strain) (engineering strain) are shown exemplarily for the flat films in the machine direction. They illustrate the wide range of mechanical responses on cold drawing for varying blend compositions. The PBSA film’s σ– ε curve shows the typical appearance of a soft and semicrystalline thermoplastic material with a yield stress $\sigma_{Y}=$ 17.5 ± 0.1 MPa and yield strain $\varepsilon_{Y}=$ 14.0 ± 0.3%, followed by strain hardening and an elongation at break $\varepsilon_{Y}=$ 760 ± 70%. On the contrary, PLA is hard and brittle with a yield point at $\sigma_{Y}=$ 59.7 ± 1.9 MPa/$\varepsilon_{Y}=$ 2.1 ± 0.1%, immediately followed by the break of the film specimen at 2.6% (see inset of Figure 7).

**Figure 8:**
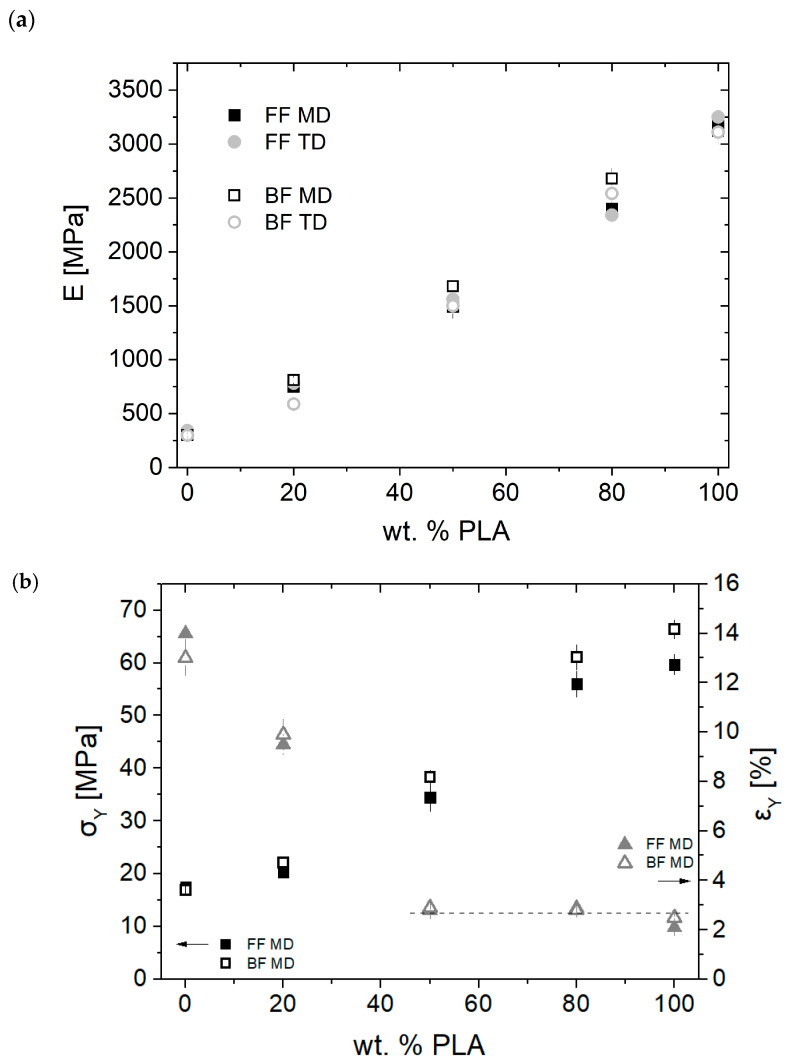
(**a**) Young’s modulus $E_{t}$ and (**b**) yield stress and yield strain from tensile measurements for flat films and blown films as a function of PLA content.

The Young’s modulus $E_{t}$ from the tensile tests varies with blend composition, and it was evaluated for all the film samples in the machine (MD) and transverse direction (TD), as depicted in Figure 8a. The general trend is nearly linear, and $E_{t}$ increases for increasing PLA content, as expected for an immiscible blend system. Whereas for the virgin PBSA and PLA $E_{t}$ is essentially independent of the film fabrication method and of the direction of testing, slight differences appear in the blends. For the blown films, the values in MD are larger than in TD; however, for the flat films, they nearly overlap.

For comparison, typical mechanical properties reported for commodity polyolefins such as LDPE and HDPE include yield stresses in the range of approximately 10–30 MPa, Young’s moduli between about 200 and 1500 MPa, and elongations at break up to 2500% [28]. The PBSA/PLA blend films investigated in this work cover this property space and partly extend beyond it. Yield stresses between approximately 15 and 65 MPa and Young’s moduli ranging from about 200 up to 3000 MPa were obtained, depending on blend composition and processing route. In contrast, the elongation at break is significantly reduced compared to polyolefins, reflecting the contribution of the PLA phase and the immiscible blend morphology.

The yield stress and yield strain depicted in Figure 8b only for the MD also show a pronounced dependence on the blend composition. Interestingly, the yield strain for virgin PBSA and PBSA-80 (>9%) is dominated by the PBSA contribution, i.e., a ductile behavior is observed. For a content of 50% of or lower, $\varepsilon_{Y}$ is practically constant at 2 to 3%, i.e., it is dominated by the PLA contribution.

### 3.3. Thermomechanical Characterization During Heating

**Figure 9:**
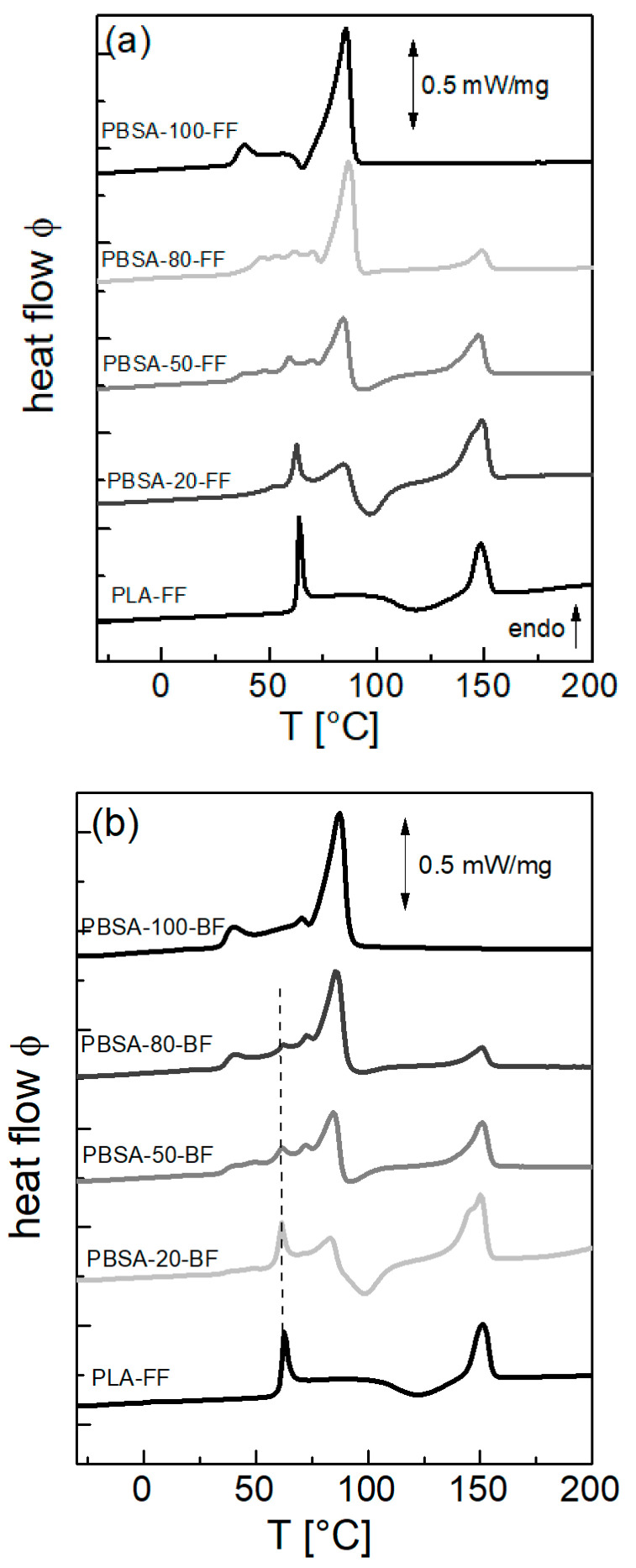
DSC heating scans of the (**a**) flat films and the (**b**) blown films with a heating rate of 10 K/min. For clarity, the heating curves are shifted vertically. The vertical dotted line in (**b**) is at the position of the enthalpy relaxation of PLA and for guidance of the eye.

DSC measurements were used to correlate the processing-induced morphology with semicrystalline structure and thermal transitions of the films. DSC scans during the first heating of the flat and blown films are shown in Figure 9. They indicate a semicrystalline state of pure PBSA in the flat and the blown films, visible as the broad melting endotherm in the temperature range between 25 °C and 95 °C. The first endotherm with a peak at around 38 °C corresponds to the melting of crystallites formed during processing when the extruded polymer melt from $T_{melt}$ was quickly cooled. Final melting takes place at 88 °C. The DSC findings in pure PBSA films are in accordance with the X-diffraction results (Figure 6). For PBSA flat films, however, an exothermic event at 64 °C is observable, indicating that a small amount of material cold-crystallized during heating. Multiple melting peaks during heating were reported for PBSA and PBS, which were assigned to crystals formed during crystallization and also to ongoing melting and recrystallization during heating [16]. Here, for PBSA-FF, the melting enthalpy of ${∆H}_{m}=$ 54 J/g was slightly lower than for PBSA-BF, which was 62 J/g. Using the melting enthalpy of an infinitely large PBS crystal ${∆H}_{m}^{\infty }=\;$183 J/g [29] and the relation $x_{c}\;[\%]=\;{∆H}_{m}/{∆H}_{m}^{\infty }\;$× 100, these values correspond to crystallinities of 30% for PBSA-FF and 34% for PBSA-BF. Note, although only available for PBS, the literature value ${∆H}_{m}^{\infty }$ is based on combined NMR, SAXS and DSC measurements [29]. To our knowledge, no experimental attempt has been made to determine ${∆H}_{m}^{\infty }$ for PBSA, although theoretical calculations have been used. However, these values for ${∆H}_{m}^{\infty }$ are about a factor of two smaller than the value used here, and therefore the PBSA crystallinity would have been overestimated [8,30]. For a PBSA with an adipate content of 25 mol.%, a crystallinity of 27% was determined from wide-angle X-ray diffraction [30], which is in good accordance with the values determined here.

In contrast to PBSA and in accordance with the data presented above, the PLA remains amorphous in the processed films. The glass transition temperature of pure PLA is visible as a pronounced step in the heat flow at $T_{g}^{PLA}=\;$61 °C, with a sharp endothermic peak on top due to enthalpy relaxation. At about 118 °C, the broad exothermic signal is attributed to cold crystallization followed by the melting endotherm, with the peak maximum at $T_{m}^{PLA}=$ 148 °C. The total melting enthalpy for integrating the exothermic and endothermic signals is zero (i.e., no crystallinity is present in the bulk sample). As Androsch et al. reported, the critical cooling rate to completely suppress crystallization is 3 K/min for a PLLA with 4% D-isomer co-units in the chain [31]. Certainly, a higher cooling rate can be assumed during solidification of the PLA films. Furthermore, during cooling from the melt at 10 K/min, the samples PLA-FF and PLA-BF only featured a glass transition step at about 51 °C (see SI Figure S1).

The DSC traces of the films containing the PBSA/PLA blends show all the signatures described for the pure polymers, but now overlapping. In the temperature range of PBSA melting, the enthalpy relaxation at the $T_{g}$ of PLA can be seen (Figure 9b). After the melting peak of PBSA, cold crystallization of PLA occurs at about 90 to 100 °C, which is a shift of 20 to 30 K to lower temperatures compared to the pure PLA film. We attribute this observation to a nucleating effect of the PBSA crystals on PLA.

**Figure 10:**
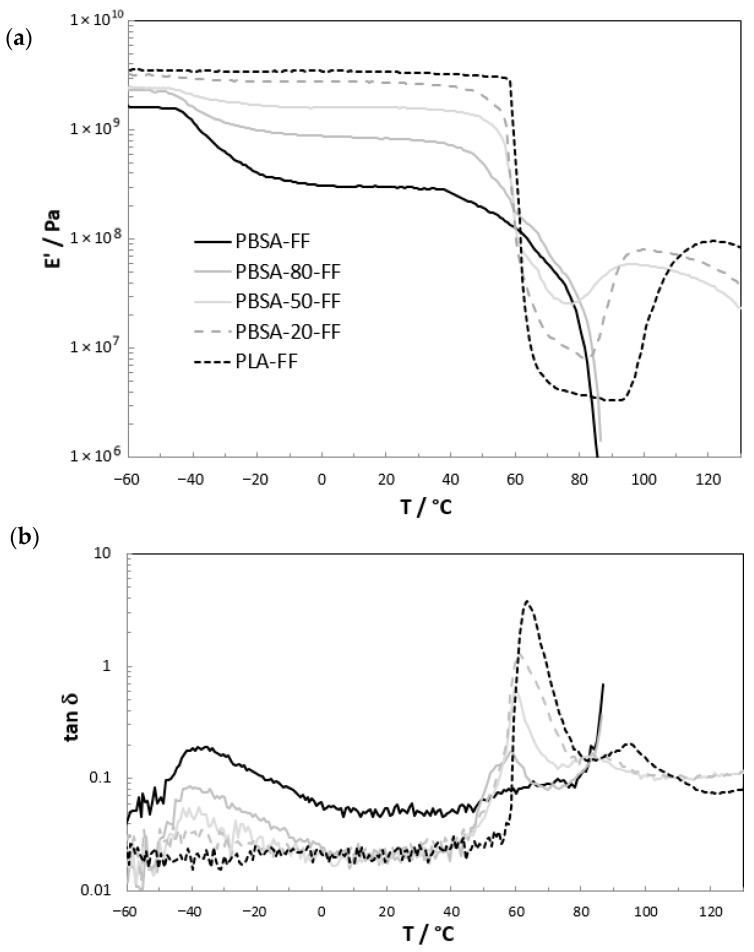
DMA heating scans for the flat films at a heating rate of 4 K/min. The (**a**) real part of Young’s modulus $E^{\prime }(T)$ and (**b**) the loss factor tan δ. The absolute values of $E^{\prime }(T)$ were vertically shifted according to the values from the tensile tests.

Complementary information to DSC was obtained for the flat films, with DMA measurements shown in Figure 10. DMA is more sensitive with respect to the glass transition region due to the loss of mechanical strength at this thermal event. The glass transition temperature of PBSA is $T_{g}^{PBSA}=-$40 °C, as visible from the drop of the real part of the modulus in Figure 10a and the peak maximum of the loss factor (Figure 10b). It remains constant for all blend compositions and is in acceptable accordance with the DSC values for non-commercial PBSA with 20 to 25 mol.% adipate comonomer [8,30].

## 4. Conclusions

In this work, commercially available poly(butylene succinate-co-adipate) (PBSA), poly(lactic acid) (PLA) and blends thereof at three different compositions were investigated. The blends were prepared by mixing the polymeric components in a co-rotating twin-screw extruder. All five materials were processed into flat and blown films using conventional extrusion equipment, thus ensuring industrial relevance of the processing conditions.

Multivariate confocal Raman microscopy (CRM) is a suitable tool for investigating the blend morphology. As revealed by CRM, the blend morphology of the flat films exhibits lamellar-like domains formed during uniaxial stretching and rapid cooling. In flat films, these lamellar domains are preferentially oriented parallel to the machine direction, whereas blown films exhibit no pronounced preferential orientation as a consequence of biaxial deformation. Typical interlamellar distances are in the low micrometer range. In contrast to CRM, with scanning electron microscopy (SEM) it is challenging to unambiguously reveal the blend morphology due to overlaying effects from sample preparation and the intrinsic inability to distinguish between phases with different chemical compositions. Complementarily, X-ray diffraction measurements in transmission showed the semicrystalline order. While PBSA is crystalline, PLA remains amorphous after the processing step, which holds both for the pure materials as well as the blends. This finding is consistent with the results from differential scanning calorimetry (DSC) from first heating and subsequent cooling, as well as dynamic mechanical analysis (DMA).

The investigated PBSA/PLA blend films span a broad range of mechanical properties, covering and partly exceeding the stiffness and strength ranges typically reported for commodity polyolefins, while exhibiting reduced ductility due to the immiscible blend morphology.

The results demonstrate how industrially relevant flat and blown film processing directly governs microphase morphology, crystalline orientation, and the resulting mechanical response of PBSA/PLA films. Due to its high relevance within the emerging bioplastics sector, PBSA will play an important role in blends with PLA for flexible film applications and related flexible packaging applications. The resulting blend morphology can be effectively analyzed using CRM in combination with complementary analytical techniques. Future work may extend this approach to related biodegradable polyester systems and to other thermoplastic processing routes, with particular emphasis on film-based applications and processing-induced orientation effects.

## Figures and Tables

**Table 1:** Melt mass temperature $T_{melt}$ at the die during processing of flat films (FF) and blown films (BF), film thickness $t_{film}$ and surface roughness $R_{a}$ in transverse direction.

Sample Name	$T_{melt}$ [°C]	$t_{film}$ [µm]	$R_{a}$ [nm]
PBSA-FF	142	71 ± 3	39 ± 6
PBSA-80-FF	190	89 ± 9	420 ± 49
PBSA-50-FF	194	79 ± 5	223 ± 35
PBSA-20-FF	187	86 ± 2	131 ± 9
PLA-FF	183	85 ± 6	28 ± 7
PBSA-BF	144	81 ± 5	20 ± 5
PBSA-80-BF	190	93 ± 10	268 ± 41
PBSA-50-BF	194	82 ± 7	282 ± 38
PBSA-20-BF	187	81 ± 7	105 ± 16
PLA-BF	183	55 ± 4	5 ± 2

## Data Availability

The original contributions presented in this study are included in the article/Supplementary Material. Further inquiries can be directed to the corresponding author.

## Supplementary Materials

The following supporting information can be downloaded at: https://www.mdpi.com/article/10.3390/polym18060761/s1, Figure S1: DSC cooling scans.

## References

[1] Tserki V., Matzinos P., Pavlidou E., Vachliotis D., Panayiotou C. (2006). Biodegradable aliphatic polyesters. Part I. Properties and biodegradation of poly(butylene succinate-co-butylene adipate). Polym. Degrad. Stab..

[2] Xu J., Guo B.-H. (2010). Poly(butylene succinate) and its copolymers: Research, development and industrialization. Biotechnol. J..

[3] Dessie W., Zhang W., Xin F., Dong W., Zhang M., Ma J., Jiang M. (2018). Succinic acid production from fruit and vegetable wastes hydrolyzed by on-site enzyme mixtures through solid state fermentation. Bioresour. Technol..

[4] Bozell J.J., Petersen G.R. (2010). Technology development for the production of biobased products from biorefinery carbohydrates—the US Department of Energy’s “Top 10” revisited. Green Chem..

[5] Skoog E., Shin J.H., Saez-Jimenez V., Mapelli V., Olsson L. (2018). Biobased adipic acid—The challenge of developing the production host. Biotechnol. Adv..

[6] Debuissy T., Pollet E., Avérous L. (2017). Synthesis and characterization of biobased poly(butylene succinate-ran-butylene adipate). Analysis of the composition-dependent physicochemical properties. Eur. Polym. J..

[7] Ahn B.D., Kim S.H., Kim Y.H., Yang J.S. (2001). Synthesis and characterization of the biodegradable copolymers from succinic acid and adipic acid with 1,4-butanediol. J. Appl. Polym. Sci..

[8] Pérez-Camargo R.A., Fernández-d’Arlas B., Cavallo D., Debuissy T., Pollet E., Avérous L., Müller A.J. (2017). Tailoring the Structure, Morphology, and Crystallization of Isodimorphic Poly(butylene succinate-ran-butylene adipate) Random Copolymers by Changing Composition and Thermal History. Macromolecules.

[9] Fujimaki T. (1998). Processability and properties of aliphatic polyesters, ‘BIONOLLE’, synthesized by polycondensation reaction. Polym. Degrad. Stab..

[10] Yoshikawa K., Ofuji N., Imaizumi M., Moteki Y., Fujimaki T. (1996). Molecular weight distribution and branched structure of biodegradable aliphatic polyesters determined by s.e.c.-MALLS. Polymer.

[11] Hallstein J., Gomoll A., Lieske A., Büsse T., Balko J., Brüll R., Malz F., Metzsch-Zilligen E., Pfaendner R., Zehm D. (2021). Unraveling the cause for the unusual processing behavior of commercial partially bio-based poly(butylene succinates) and their stabilization. J. Appl. Polym. Sci..

[12] Park J.W., Im S.S. (2002). Phase behavior and morphology in blends of poly(L-lactic acid) and poly(butylene succinate). J. Appl. Polym. Sci..

[13] Yokohara T., Yamaguchi M. (2008). Structure and properties for biomass-based polyester blends of PLA and PBS. Eur. Polym. J..

[14] Deng Y., Thomas N.L. (2015). Blending poly(butylene succinate) with poly(lactic acid): Ductility and phase inversion effects. Eur. Polym. J..

[15] Lee S., Lee J.W. (2005). Characterization and processing of Biodegradable polymer blends of Characterization and processing of Biodegradable polymer blends of poly(lactic acid) with poly(butylene succinate adipate). Korea-Aust. Rheol. J..

[16] Wang Y., Mano J.F. (2007). Biodegradable poly(L-lactic acid)/poly(butylene succinate-*co*-adipate) blends: Miscibility, morphology, and thermal behavior. J. Appl. Polym. Sci..

[17] Ojijo V., Cele H., Sinha Ray S. (2011). Morphology and Properties of Polymer Composites Based on Biodegradable Polylactide/Poly[(butylene succinate)-*co*-adipate] Blend and Nanoclay. Macro Mater. Eng..

[18] Yu P., Li S., Wei Z., Peng C., Cao N., Wan C., Bi S., Chen X. (2023). In-situ generation of biodegradable poly(lactic acid)/poly(butylene succinate) nanofibrillar composites via a facile and cost-effective strategy of pressure-induced flow processing. Polym. Adv. Technol..

[19] Navarro-Baena I., Sessini V., Dominici F., Torre L., Kenny J.M., Peponi L. (2016). Design of biodegradable blends based on PLA and PCL: From morphological, thermal and mechanical studies to shape memory behavior. Polym. Degrad. Stab..

[20] Benito-González I., Martínez-Sanz M., López-Rubio A., Gómez-Mascaraque L.G. (2020). Confocal Raman imaging as a useful tool to understand the internal microstructure of multicomponent aerogels. J. Raman Spectrosc..

[21] Liu X.-Y., Guo S., Bocklitz T., Rösch P., Popp J., Yu H.-Q. (2022). Nondestructive 3D imaging and quantification of hydrated biofilm matrix by confocal Raman microscopy coupled with non-negative matrix factorization. Water Res..

[22] Malz F., Arndt J.-H., Balko J., Barton B., Büsse T., Imhof D., Pfaendner R., Rode K., Brüll R. (2021). Analysis of the molecular heterogeneity of poly(lactic acid)/poly(butylene succinate-co-adipate) blends by hyphenating size exclusion chromatography with nuclear magnetic resonance and infrared spectroscopy. J. Chromatogr. A.

[23] Eilers P.H., Boelens H.F. (2005). Baseline Correction with Asymmetric Least Squares Smoothing. Leiden Univ. Med. Cent. Rep..

[24] Pedregosa F., Varoquaux G., Gramfort A., Michel V., Thirion B., Grisel O., Blondel M., Prettenhofer P., Weiss R., Dubourg V. (2011). Scikit-learn: Machine learning in Python. J. Mach. Learn. Res..

[25] (2003). Plastics—Determination of Tensile Properties. Part 3: Test Conditions for Films and Sheets.

[26] Sachs N.W. (2005). Understanding the surface features of fatigue fractures: How they describe the failure cause and the failure history. J. Fail. Anal. Preven..

[27] Nofar M., Tabatabaei A., Sojoudiasli H., Park C.B., Carreau P.J., Heuzey M.-C., Kamal M.R. (2017). Mechanical and bead foaming behavior of PLA-PBAT and PLA-PBSA blends with different morphologies. Eur. Polym. J..

[28] Phillips P.J., Patel J. (1978). The influence of morphology on the tensile properties of polyethylenes. Polym. Eng. Sci..

[29] Yu Q., Anuar A., Petzold A., Balko J., Saalwächter K., Thurn-Albrecht T. (2023). The Semicrystalline Morphology of Polybutylene Succinate Supports a General Scheme Based on Intracrystalline Dynamics. Macromol. Chem. Phys..

[30] Nikolic M.S., Djonlagic J. (2001). Synthesis and characterization of biodegradable poly(butylene succinate-co-butylene adipate)s. Polym. Degrad. Stab..

[31] Androsch R., Di Lorenzo M.L., Schick C. (2016). Crystal nucleation in random l/d-lactide copolymers. Eur. Polym. J..

