# Supplementary material for: From Film Processing to Microphase Orientation: Structure–Property Relationships in Commercial PBSA/PLA Blend Films
Source: Polymers (Basel). 2026 Mar 20;18(6):761. doi: 10.3390/polym18060761 (PMC13029859; doi:10.3390/polym18060761)
Supplement: Supplementary file 1 [file polymers-18-00761-s001.zip › polymers-3531552-supplementary.pdf]

Supporting Information

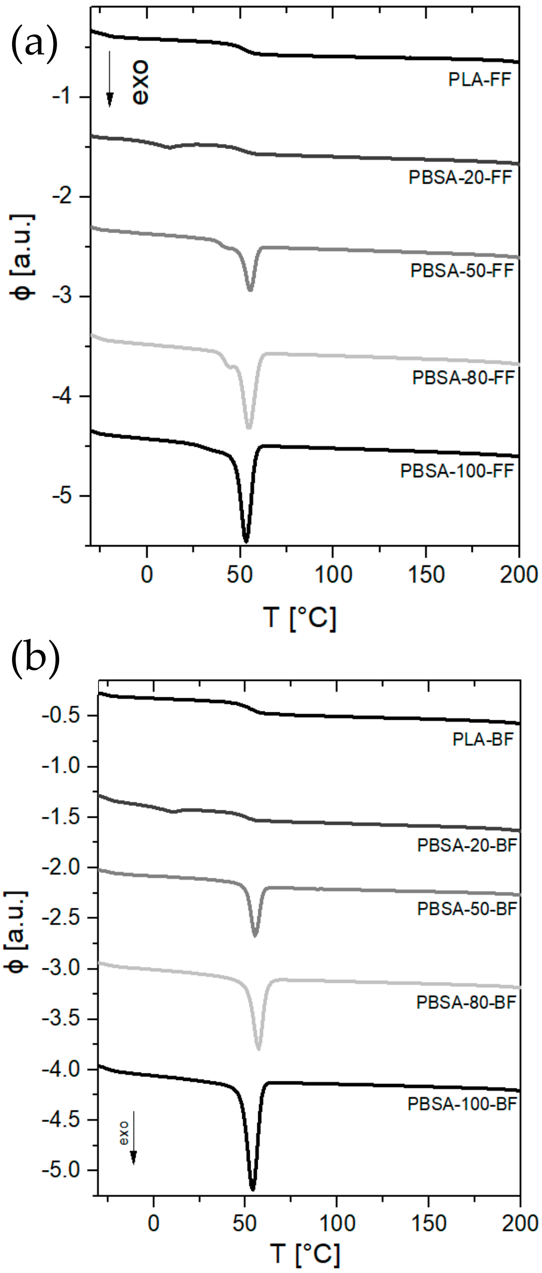

Fig. S1: DSC cooling scans of the (a) flat films and the (b) blown films with a cooling rate of 10 K/min. For clarity, the cooling curves are shifted vertically.
